# Long-term outcome following medial open reduction in developmental dysplasia of the hip: a retrospective cohort study

**DOI:** 10.1007/s11832-016-0729-5

**Published:** 2016-04-15

**Authors:** Richard O. E. Gardner, Catharine S. Bradley, Om P. Sharma, Lin Feng, Michelle EyunJung Shin, Simon P. Kelley, J. H. Wedge

**Affiliations:** CURE Ethiopia Children’s Hospital, Addis Ababa, Ethiopia; Department of Rehabilitation Services, The Hospital for Sick Children, 555 University Avenue, Toronto, ON M5G 1X8 Canada; Division of Orthopaedic Surgery, The Hospital for Sick Children, 555 University Avenue, Toronto, ON M5G 1X8 Canada; Department of Orthopaedic Surgery, Xiamen Woman and Children’s Hospital, Xiamen, China; Department of Surgery, Mt. Sinai Hospital, Toronto, Canada

**Keywords:** Developmental dysplasia of the hip, Hip dislocation, Medial open reduction, Avascular necrosis

## Abstract

**Introduction:**

Avascular necrosis (AVN) is a serious complication of treatment for developmental dysplasia of the hip. There is ongoing controversy regarding AVN and its influence on hip development following medial open reduction (MOR).

**Purpose:**

The aim of our study was to (1) determine the long-term prevalence of AVN following MOR, (2) evaluate hip development after MOR, and (3) identify predictors of AVN and radiographic outcome at skeletal maturity after MOR.

**Methods:**

A retrospective cohort analysis of 60 patients (70 hips) who underwent MOR with a mean follow-up of 10.83 years (5.23–16.74) was conducted. AVN was recorded according to Bucholz and Ogden classification and radiographic outcome based on Severin grading. AVN and hip morphology related to length of follow-up were evaluated. Chi-squared and *t*-tests were used to identify relationships between AVN and other variables. Logistic regression was used to assess predictors of AVN and Severin outcome.

**Results:**

The rate of clinically significant AVN (types 2–4) following MOR was 32.9 % with type 2 accounting for 82.6 % of these cases. While early acetabular development was satisfactory, long-term outcome was unsatisfactory in 26 % of cases with AVN (vs 8.7 % of cases without AVN). A higher rate of AVN was identified when hips were immobilized in ≥60° of abduction postoperatively. A higher rate of poor Severin outcome was identified in hips with AVN.

**Conclusions:**

Our findings suggest that there is a high rate of AVN and unsatisfactory long-term outcome following MOR. AVN remains a significant concern following MOR surgery for developmental dysplasia of the hip that may not be apparent until long-term evaluation.

## Introduction

Developmental dysplasia of the hip (DDH) in infancy spans a wide spectrum of pathology from the hip that is clinically stable but abnormal on ultrasound, to the dislocated irreducible hip. While the Pavlik harness is the most common treatment for DDH in the neonate [1–3], some hips fail to stabilize and surgical treatment is required. However, the optimal surgical approach for the irreducible hip in the infant and its timing remain controversial [4, 5].

The decision on which approach to use may be determined in part by the risk profile of each intervention. Avascular necrosis (AVN) is considered to be the most significant complication following treatment for DDH because of the consequent early onset of progressive osteoarthritis, disability and the need for total hip arthroplasty at a young age [6]. It is therefore important to understand the risk of AVN and its long-term implications for each management strategy.

Medial open reduction (MOR) is an established technique that allows direct access to the blocks to hip reduction and does not violate the hip abductors or iliac apophysis. The resulting scar is cosmetically acceptable and bilateral hip dislocations may be addressed with minimal blood loss [5, 7–9]. The primary indication for MOR at our institution has been a hip that had Pavlik harness failure or was thought to be irreducible by closed means. The primary concern with MOR is the inherent vulnerability of the medial femoral circumflex artery and the risk of AVN that can arise from ligation or damage to this vessel [10, 11]. Case series reports of MOR have demonstrated rates of AVN ranging from 0−67 % [12, 13]. More recently, a systematic review of MOR reported a rate of AVN of 24 % at skeletal maturity and hips with AVN had a statistically higher rate of unsatisfactory outcome at skeletal maturity based on Severin grading [14].

The purpose of this study was

1. To determine the long-term prevalence of AVN following MOR.
2. To evaluate hip development after MOR.
3. To identify predictors of AVN and radiographic outcome at skeletal maturity after MOR.

## Methods

This study was a retrospective cohort analysis of patients who had undergone MOR at our institution between 1988 and 2005. Ethical approval was obtained through our institution’s research ethics board. Inclusion criteria for this study were patients who had undergone MOR for DDH with a minimum follow-up of 5 years and radiographs at final follow-up. In each case, the diagnosis of hip dislocation was confirmed on ultrasound or radiographic findings. Exclusion criteria for this study were patients with a diagnosis of neuromuscular disease, teratologic hip dislocation, or skeletal dysplasia.

### Surgical technique

A transverse incision was performed 1 cm distal to the groin crease, overlying the adductor longus tendon. The fascia was split longitudinally and the adductor longus tendon was divided close to the insertion. The Ludloff interval was used between adductor brevis and pectineus. The medial femoral circumflex vessels were isolated in a vascular sling. A psoas tenotomy was performed and the capsule opened with a T-shaped capsulotomy. The transverse acetabular ligament and ligamentum teres were divided. The hip was then reduced under direct view. A spica cast was applied in the human position of 100 °flexion, <50° of abduction and <10° of internal rotation as described by Salter [15]. All patients were immobilized in this position for a minimum of 3 months either by casting with an interval change or by a combination of sequential casting and bracing. Postoperative computed tomography (CT) or magnetic resonance imaging (MRI) was used to confirm reduction and the hip abduction angle was measured from the axial slice of the MRI or CT. The hip abduction angle was measured from the axis of the femoral shaft to a line perpendicular to the horizontal line passing through both posterior superior iliac spines.

### Outcome measures

Pelvic radiographs were evaluated at 1 and 4 years and at final follow-up post-surgery. AVN was graded according to Bucholz and Ogden classification [16]. Type 1 AVN describes hips that have temporary delay/irregular ossification, with minimal residual deformity and is typically considered not clinically significant [9, 17, 18]. The acetabular index was measured until closure of the triradiate cartilage and the Sharp angle [19] was measured thereafter. The center-edge angle (CEA) of Wiberg [20] and the Severin grade [21] were recorded at final follow-up. Severin grade I-II radiological outcome is generally considered satisfactory and Severin III-VI unsatisfactory [5, 9, 17, 22]. Other recorded variables included preoperative interventions and secondary procedures following MOR.

### Statistical analysis

Statistical analysis was undertaken using SPSS (Version 21). Sample characteristics including gender, age at MOR, previous treatment, bilateral versus unilateral involvement, further surgery, re-dislocation, position of immobilization, length of follow-up and Severin grade at final follow-up were assessed using descriptive statistics. The overall AVN rate, type of AVN rates and AVN related to length of follow-up in years were evaluated. Acetabular morphology was also assessed in relation to length of follow-up. Chi-squared testing was used to determine if there were relationships between AVN, Severin grade, re-dislocation, gender, side of dislocation, bilaterality, need for further surgery and previous treatment. *T*-tests were used to evaluate the relationships between age at MOR, the degree of hip abduction in spica, age at further surgery, age at follow-up, length of follow-up, AVN and Severin grade at final follow-up. Statistically significant variables (*p* < 0.05) or borderline significant variables (*p* = 0.05–0.10) were assessed for collinearity and entered into a logistic regression model to assess if any of these variables were predictive of AVN or final Severin grade.

## Results

A total of 70 hips in 60 patients underwent MOR, with the majority being female (47/60). Most had failed treatment preoperatively—70 % (42/60) with a Pavlik harness, 7 % (4/60) with a combination of Pavlik harness and traction, and 3 % (2/60) with preoperative traction only. CT or MRI scans in the postoperative hip spica were available for 90 % of the hips (63/70). The mean angle of hip abduction in spica was 65.8° (45°–86°). The mean age at MOR was 6.8 months (1.6–17.5 months). The mean length of follow-up was 10.83 years (5.23–16.74 years).

### Prevalence of AVN

**Fig. 1:**
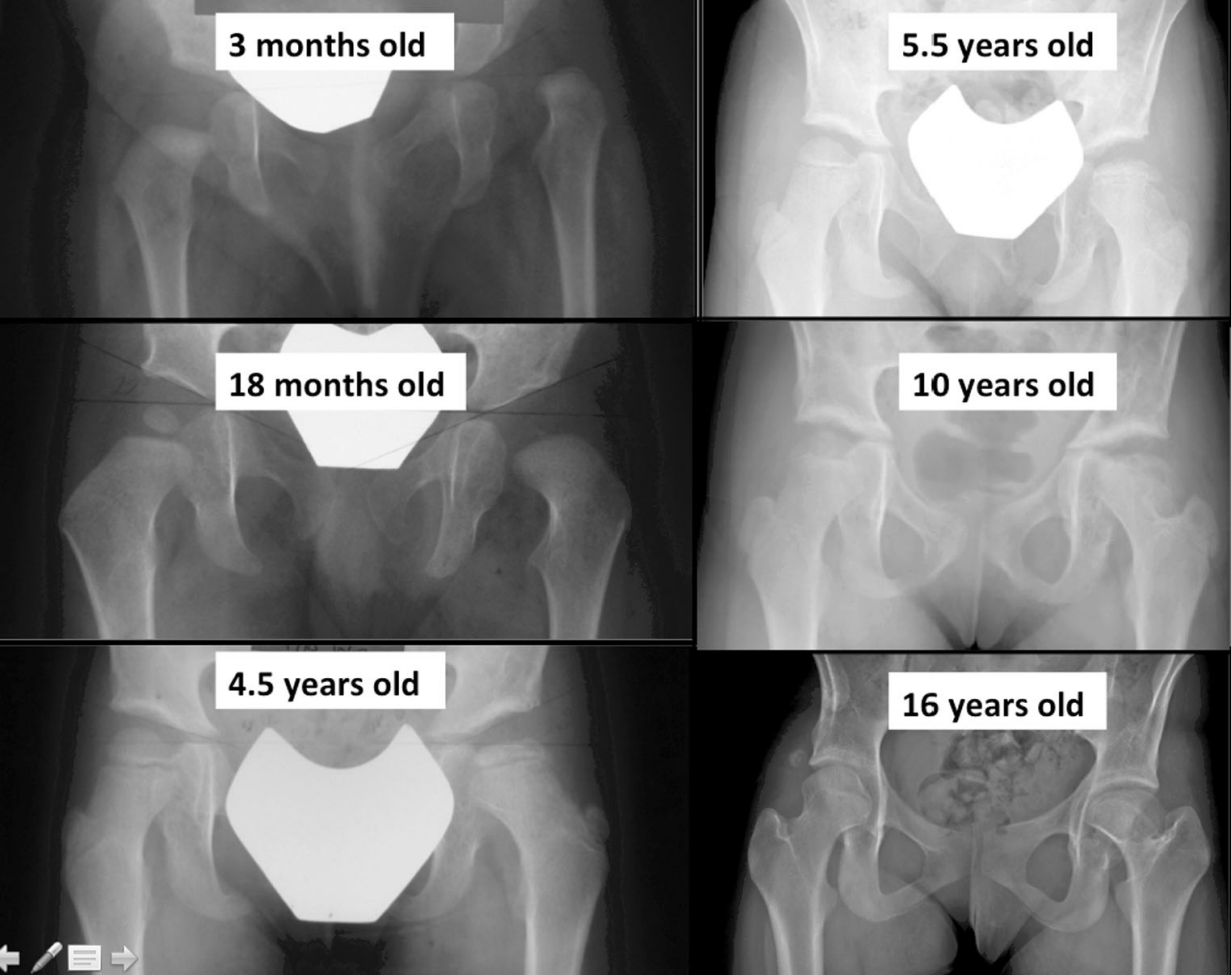
Example of type 2 AVN development. There is persistent asymmetry in the ossific nucleus, but the reversal of the physeal inclination becomes apparent at 5 years of age. The caput valgus deformity is noted at 10 years

The rate of clinically significant AVN (types 2–4) was 32.9 % (23/70) at final follow-up. Of these, 82.6 % (19/23) were type 2 AVN. The prevalence of AVN and in particular, type 2 AVN increased with longer follow-up. The data are presented in Table 1. An example of type 2 AVN development is depicted in Fig. 1.

**Table 1:** Prevalence of AVN in relation to years of follow-up

Years following surgery	Hips (*n*)	Overall AVN rate (%)	Type 2 AVN (%)	Type 2 AVN/all AVN (%)
>5	70	32.9	27.1	82.6
>8	57	35.1	28.1	80.0
>12	29	37.9	31.0	81.2
>15	7	57.1	57.1	100

### Hip development and secondary surgeries

**Fig. 2:**
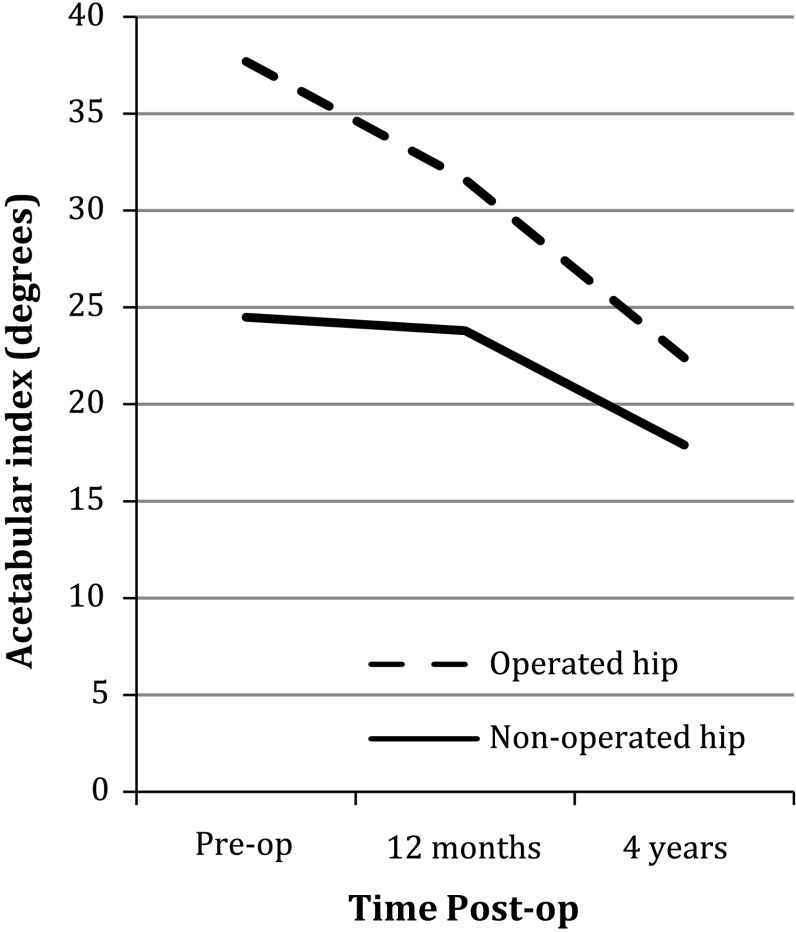
Progression of acetabular index following surgery

There was a rapid improvement in acetabular morphology in the first year after surgery. This improvement continued in a linear fashion to 4 years postoperatively (Fig. 2). The mean CEA for the treated hips (29.4°) was within the normal range at final follow-up and was comparable to the mean angle for the contralateral hips (CEA 32.7°).

Secondary surgery was required in 28.6 % of all hips (20/70). The details of these surgeries in relation to AVN are presented in Table 2. The overall rates of secondary surgery were not significantly different between hips with AVN (30.4 %) and hips without AVN (27.7 %). However, those hips that did have AVN required more combined acetabular and femoral procedures (17.4 % of hips with AVN vs 0 % of hips without AVN).

**Table 2:** Secondary surgeries following MOR

	MOR without AVN	MOR with AVN
Redislocation requiring		
Closed reduction	1/47	0/23
Open reduction ± Salter	2/47	2/23
Further surgery		
Salter alone	5/47	1/23
VDRO alone	5/47	0/23
Salter and VDRO	0/47	4/23

*VDRO* Varus derotation osteotomy

### Predictors of AVN and radiographic outcome

The only two variables found to have a statistically significant relationship with AVN were age at MOR (with a higher incidence of AVN noted in children undergoing MOR >12 months of age, *p* = 0.045) and degree of hip abduction in spica (*p* = 0.048). However, logistic regression demonstrated that while age at MOR was not shown to be a significant predictor of AVN, immobilization ≥60° of abduction was predictive with an odds ratio of 1.12 (95 % confidence interval [CI] 1.02–1.24; *p* = 0.025). On further analysis, significant AVN was found in 36.5 % (19/52) of hips immobilized in >60° of abduction, compared to 0 % (0/11) of hips immobilized in <60° of abduction (*p* = 0.016).

There were six re-dislocations, all of which occurred in hips immobilized in >60° of abduction. All were in female patients with age at surgery 2.8–14.9 months (average 7.4 months). Two of these hips developed significant AVN (both type 4).

The presence of AVN resulted in a significantly poorer outcome based on Severin grading. Severin grade of ≥3 was found in 26 % (6/23) of hips with AVN, compared to 8.5 % (4/47) in hips without AVN (*p* = 0.048). Other variables found to have a statistically significant relationship with Severin grade were age at follow-up (*p* = 0.02), length of follow-up (*p* = 0.02) and treatment prior to MOR (*p* = 0.04). Regression analysis demonstrated that in this cohort only the presence of significant AVN (types 2–4) was predictive of unsatisfactory Severin outcome with an odds ratio of 6.44 (95 % CI 1.10–22.54; *p* = 0.039).

## Discussion

This study sought to determine the long-term prevalence of AVN following MOR, to evaluate hip development after MOR including the need for secondary surgeries, and to identify predictors of AVN and radiographic outcome at skeletal maturity after MOR. Our study has a number of limitations. First, it is retrospective and accordingly, some outcomes are reliant on the accuracy of the medical records. However, the primary outcomes of this study, including AVN, Severin grading and acetabular morphology were evaluated by the authors from original medical imaging (radiographs, CT or MRI). Second, despite a mean follow-up of 10 years, not all the patients were followed until skeletal maturity. As such, given the trend of increasing rates of clinically significant AVN with longer follow-up, we may, in fact, be underestimating the prevalence of AVN post-MOR in this study, as type II AVN may not present radiographically until 8 or 9 years of age. However, we still consider the rates of AVN found in this study to be unacceptably high. Third, while the reliability and validity of CT and MRI have not been confirmed for evaluating the position of immobilization in spica, we consider these methods more plausible than clinical evaluation. Further assessment of this measurement, including standardization of patient position, reliability and validity is warranted in order to fully understand the impact of immobilization position on the development of AVN.

The overall AVN rate in this study was 32.9 % at final follow-up with type 2 AVN accounting for the majority of these cases. Type 2 AVN is the most pernicious of the growth disturbances seen following surgery for DDH, typically becoming symptomatic with pain in teenage years and leading to early degenerative changes in the hip joint. Surgical intervention, incorporating proximal femoral and acetabular osteotomies, is frequently required to restore a normal biomechanical environment for the hip. This pattern of growth disturbance is often delayed in its presentation, typically identifiable radiographically between 5 and 12 years postoperatively [6, 23, 24]. For this reason, the minimum follow-up in our study was 5 years in an attempt to identify AVN that is not apparent in a cohort with limited follow-up. We have demonstrated that the prevalence of AVN increases strikingly towards skeletal maturity and in those with >15 years of follow-up (*n* = 7), the AVN rate was 57 %, all being type 2. Again, this finding suggests that we may have underestimated the total incidence of type 2 AVN and its impact on final hip outcome according to Severin grade.

We noted an early improvement in the acetabular index following MOR surgery. Notably, the mean trajectory is in agreement with Severin I-II outcome according to Albinana et al. [25]. In unilateral cases, the contralateral acetabular index demonstrated normal progression. The most common secondary procedure was the Salter osteotomy. Whilst the presence of AVN had no bearing on the rates of secondary surgery, AVN did affect the type of surgery required. In cases with AVN, 17.4 % (4/23) required a combined femoral and acetabular procedure, compared to 0 % (0/47) when there was no evidence of AVN. This suggests a concerning trend that more extensive reconstructive surgery is required in the face of AVN.

In this cohort, immobilization in spica of ≥60° of abduction was the only significant predictor of clinically significant AVN. Many authors have noted that extreme abduction correlates with AVN [22, 26–29], a finding supported by the results of our study. It was interesting to note that all the redislocations also occurred in the hips with an abduction angle in spica of 60°. This is an important finding as there may be a subset of hips that are too inherently unstable for treatment by MOR, requiring extreme positioning in a spica in an attempt to prevent redislocation, but concomitantly increasing the risk of AVN.

The majority of hips in this study were treated preoperatively in a Pavlik harness while only a few were treated in traction. However, treatment prior to MOR was not found to be associated with the development of AVN. This finding is consistent with other studies reporting a very low rate of AVN following judicious Pavlik harness use [2, 30], but is in contrast with a recent publication suggesting that previous Pavlik harness use significantly increases the risk of AVN in hips that subsequently require closed or open reduction [31]. There are several theories as to the cause of AVN following MOR. Iatrogenic injury to the medial femoral circumflex artery is frequently postulated, although several authors have reported no correlation from cases that required ligation of the artery [9, 32–34].

In reality, AVN is only a concern if it results in a clinically poor outcome for the patient. Severin III-VI hips are deemed to be unsatisfactory due to the increased risk of degenerative arthritis [35, 36]. We have demonstrated that not only do hips that have undergone MOR have a very high rate of clinically significant AVN, but also that clinically significant AVN is predictive of poor Severin grade in this cohort. Based on the rates of clinically significant AVN, and the associated poor radiographic outcome identified in our analysis of this cohort, as well as on corresponding evidence in the literature at large [14] we have abandoned the use of MOR surgery at our institution.
